# Environmental noise pollution and its association with temporomandibular joint disorders, malocclusion, and bruxism: A Comparative cross-sectional study of adults in high-traffic and low- traffic areas

**DOI:** 10.1371/journal.pone.0360667

**Published:** 2026-10-09

**Authors:** Abdullah Hasan A. Alshehri, Abdulkhaliq Ali F. Alshadidi, Lujain Ibrahim N. Aldosari, Sultan Mohammed Kaleem, Vishwanath Gurumurthy, Rajesh Vyas, Sunil Kumar Vaddamanu

**Affiliations:** 1 Department of Prosthodontics, College of Dentistry, King Khalid University, Abha, Saudi Arabia; 2 Department of Oral Diagnosis, Oral Biology and Periodontology, College of Dentistry, King Khalid University, Abha, Saudi Arabia; 3 Department of Allied Dental Health Sciences, College of Applied Medical Sciences, King Khalid University, Abha, Saudi Arabia; VIT Bhopal University, INDIA

## Abstract

**Background:**

Chronic environmental noise exposure is a recognized public health concern linked to stress-mediated physiological responses, yet its specific impact on temporomandibular disorders (TMD), bruxism, and malocclusion remains poorly investigated.

**Objectives:**

To compare the prevalence of TMD, malocclusion, and bruxism between adults residing in high-traffic versus Low- traffic areas in New Delhi, India, and to identify independent predictors of TMD risk.

**Methods:**

This comparative cross-sectional study enrolled 300 adults (150 per group) from high traffic and low traffic residential areas of New Delhi, India. Individual-level noise exposure (LAeq,24h) was objectively measured. TMD was diagnosed using Diagnostic Criteria for Temporomandibular Disorders (DC/TMD Axis I criteria). Bruxism, malocclusion, perceived stress scale −1= (PSS-10), and Pittsburgh sleep quality (PSQI) were assessed using validated instruments. Multivariable logistic regression and mediation analysis were performed.

**Results:**

High-traffic residents had significantly higher TMD prevalence (61.3% vs. 25.3%), bruxism (42.7% vs. 14.0%), and malocclusion (52.0% vs. 26.0%; all p < 0.001). A significant dose-response relationship was confirmed (χ² for trend p < 0.001). High noise exposure was the strongest independent predictor of TMD (aOR = 3.10; 95% CI: 2.00–4.80). Bruxism and perceived stress accounted for 41.4% of this association in exploratory mediation analysis, a pattern compatible with mediation, though the cross sectional design precludes establishing temporal precedence.

**Conclusions:**

Environmental noise exposure was independently and dose-dependently associated with TMD andbruxism. malocclusion was also prevalent in high traffic areas; however, this cross sectional association more likely reflects pre-existing skeletal or occlusal characteristics than a causal effect of noise, and should be interpreted with caution. These findings support noise abatement policies and targeted oral health screening in high traffic urban environments.

## Introduction

One of the most pressing environmental health issues facing developing countries today is noise pollution. Noise levels in urban areas have exceeded the WHO recommended limit of 53 dBA during the daytime hours in residential settings [1,2]. Exposure to elevated environmental noise is associated with cardiovascular disease risk, sleep disturbance, as well as the dysregulation of the Hypothalamic–pituitary–adrenal (HPA) axis [3]. However, existing evidence on the impact of noise exposure on oral health outcomes is limited to a small number of studies of occupational driving-related stress and general orofacial symptoms, without objective noise measurement or validated TMD diagnostic criteria [4–9].

Temporomandibular disorders (TMD) are conditions that affect the temporomandibular joint (TMJ) and muscles involved in jaw movement. Patients who suffer from TMD commonly experience jaw pain and limitation of jaw movement, as well as clicking and popping sounds of the TMJ [10,11]. High prevalence rates and reduced quality of life have been well documented in patients with TMD. Occlusion, defined as the relationship between the upper and lower teeth when they approach each other, can be disrupted in TMD patients [10]. Occlusal interference is known to cause alterations in joint loading which can exacerbate TMD symptoms and dysfunction.

Noise could potentially play a role in the development or worsening of TMD. Prolonged noise exposure is associated with activation of the HPA axis and subsequent increases in cortisol secretion and catecholamines, and is associated with oxidative stress and vascular dysfunction [12] and can lead to heightened tension in masticatory muscles [13,14]. These physiologic changes can induce bruxism, or jaw clenching and teeth grinding, which can overload the TMJ and contribute to accelerated tooth wear [13,14]. Furthermore, road traffic noise has been shown to increase HPA activity during sleep when compared to low traffic noise [15,16]. This finding suggests that individuals exposed to traffic noise experience heightened stress responses even while sleeping.

Although these studies provide evidence that prolonged noise exposure could have deleterious effects on TMD development and severity, no studies have compared TMD prevalence, occlusal problems, and bruxism between individuals exposed to high-traffic noise and those living in low-traffic areas, utilizing both objective noise level assessments and validated diagnostic criteria [4–9].

The aim of this study was to compare the prevalence of TMD, malocclusion, and bruxism between adults living in high traffic areas versus low traffic areas and to determine whether high traffic residence is an independent predictor of TMD after controlling for psychosocial stressors. We hypothesized that residence in a high traffic areas would be independently associated with higher prevalence of TMD, bruxism, and malocclusion.

## Methodology

### Study design and setting

This comparative cross-sectional study conducted in New Delhi, India and reported in accordance with the STROBE guidelines (Supporting file 1). The city of New Delhi was chosen because it ranks among the most noise polluted capitals around the globe. Daytime road traffic noise levels along commercial and arterial corridors have been reported to exceed 70–85 dBA [17]. Participants were enrolled from one high traffic location and one low traffic location within New Delhi, India. Briefly the high traffic location was defined by sustained traffic flow and measured noise levels ≥65 dBA. The low traffic location consisted of residential neighbourhoods with near constant low-volume traffic flow and ambient noise <65 dBA. This 65 dBA cut off corresponds to the threshold established by the WHO Environmental Noise Guidelines (2018), above which road traffic noise is associated with adverse health outcomes [18].

A sampling frame of households in each area was obtained from door to door household listing conducted by the study team. Households were selected using systematic sampling with a sampling interval of every 5^th^ household, starting from a randomly selected household in each area, until the target sample size was reached. Within each selected household, one eligible adult was chosen using the kish grid method. Groups were additionally frequency matched on age and sex at the time of recruitment. Application of exclusion criteria to both groups reduced confounding by exclusion of individuals with major comorbidities affecting the TMJ. Objective noise measurement at participants residences (rather than delf reported exposure) reduce information bias. The study was conducted in accordance with the Declaration of Helsinki, and the protocol was approved by the Research Ethics Committee at King Khalid University, Saudi Arabia [KKU-11-2026-5]. The study protocol was developed, and all participants gave their written informed consent for inclusion before they participated in the study.

### Participants

Three hundred adult participants were recruited and enrolled into the study (n = 150 per group). Participants were required to be between the ages of 18–60 years old and reside at the subject address for at least two consecutive years. Participants were excluded from participation if they had previous surgery or trauma to the TMJ or craniofacial structures, neurologic disorders, were taking centrally acting drugs known to alter muscle tone, or had severe systemic disease. Groups were matched on frequency by age and sex. Educational status was self-reported and classified into two categories: “primary/secondary” (no formal schooling through completion of secondary/high school) and “tertiary” (any diploma, undergraduate, or postgraduate qualification beyond secondary school). An a priori power calculation demonstrated that 138 participants per group (276 total) were required to detect an OR of 2.0 with 80% power at α = 0.05 assuming 40% baseline prevalence of TMD. This minimum was inflated to 150 participants per group (300 total), a buffer of approximately 9%, to safeguard statistical power against minor data quality exclusions during analysis. A separate inflation for anticipated non response was not incorporated into this calculation, as prior community engagement in the target neighbourhoods indicated a very low expected refusal rate among eligible residents; this was consistent with the 100% consent rate ultimately observed among eligible participants. The larger initial approach of 412 adults (206 per area) instead reflects the abticipated proportion of individuals expected to be ineligible during door to door screening (approximately 27%, based on the ≥ 2 year residency and age criteria), rather than anticipated non participation.

Four hundred twelve adults were approached at the target neighbourhoods (206 per area). Of these, 112 were excluded for not meeting eligibility criteria (<2 years residence [n = 68]; age outside range of 18--60 years [n = 21]; previous TMJ surgery/trauma [n = 15]; other [n = 8]), leaving 300 eligible adults, all of whom consented to participate (response rate among eligible individuals = 100%). No eligible individual refused participation. Three hundred participants completed signed informed consent (150 per group) and were enrolled. Zero participants were lost to follow-up, and all 300 completed the study outcome assessments.

### Noise exposure measurement

Noise exposure was assessed at the participants’ primary residence. Ambient noise levels were recorded using a calibrated Type-2 Integrating Sound Level Meter compliant with IEC 61672−1. Measurements were made at six time points throughout day (06: 00–08:00, 09:00–11:00, 12:00–14:00, 15:00–17:00, 18:00–20:00, 21:00–22:00) while positioned 1.5 metres from the structure façade at ear height. The energy-averaged LAeq,24h was calculated and recorded for each participant per ISO 1996–2:2017 standard. Participants were assigned to either high or low noise exposure groups based on their LAeq,24h measurements. High noise exposure was defined as LAeq,24h > 65 dBA and low noise exposure was defined as LAeq,24h ≤ 65 dBA using cut points established by WHO Environmental Noise Guidelines (2018) [19].

### Clinical examination and TMD diagnosis

All clinical examinations were performed by two calibrated examiners (intra-examiner κ = 0.84, 95% CI: 0.70–0.98; inter-examiner κ = 0.79, 95% CI: 0.65–0.93). Diagnosis of TMD was made using the DC/TMD Axis I criteria [20]. Briefly, clinical evaluation consisted of standardised assessment of mandibular range of motion, joint auscultation and palpation for clicking and crepitus, bilateral extraoral and intraoral muscle palpation at standardized sites. Based on clinical findings, participants were determined to be TMD present or absent according to DC/TMD Axis I diagnostic criteria. It is noted that DC/TMD Axis I criteria incorporated both signs (e.g., crepitus clicking, limited range of motion) and symptoms (e.g., pain on palpation, pain on jaw movements) both were assessed as part of the standardised clinical examination. An additional ordinal severity grading of TMD was completed using the Helkimo Clinical Dysfunction Index (Di 0–III). DC/TMD Axis II psychosocial screening included Patient Health Questionnaire-4 (PHQ-4) a 4-item screener (2 anxiety items, 2 depression items) scored 0–3 per item, yielding a total range of 0–12; a score ≥6 was defined as indicating clinically relevant anxiety/depression, consistent with established distress thresholds. The Graded Chronic Pain Scale (GCPS) was also administered, classifying participants into five hierarchical grades (0 = no pain; I–II = low/high pain intensity with low disability; III–IV = moderate-to-severe pain-related disability) based on pain intensity and disability subscale scores. These psychosocial instruments were included as covariates in regression models.

Classification of occlusal status (Angle’s classification) was completed with malocclusion being defined as any angle classification other than class I ideal occlusion. Pain intensity in the TMJ was measured on a 100 mm Visual Analogue Scale (VAS) with scores categorized as none (o mm), mild (1–30 mm), moderate (31–60 mm), or severe (61–100 mm). Maximum unassisted mouth opening (MMO) was measured in millimetres using a ruler calibrated to the nearest millimetre, serving as an objective quantitative measure of mandibular opening. Restricted mouth opening was defined as MMO < 40 mm, according to DC/TMD criteria.

### Assessment of TMJ dysfunction severity

As noted above, an additional ordinal severity grading of TMD was completed using the Helkimo Clinical Dysfunction Index (Di 0–III), categorizing participants as having none (Di 0), mild (Di I), moderate (Di II), or severe (Di III) dysfunction. This rating system was based on clinical assessment of mandibular range of motion, TMJ function, muscle and joint pain upon palpation, as well as pain on jaw movement. The Helkimo Index was used for purposes of severity stratification and group comparison only; study participants were diagnosed with and classified according to DC/TMD Axis I criteria as described above [20]. The decision to utilize both diagnostic tools was made in order to capture both standardized diagnostic information that meets contemporary requirements for standardization in research, as well as an ordinal severity grading that could be compared to the larger epidemiological TMD literature.

### Bruxism assessment

Bruxism was evaluated according to the three-level diagnostic criteria by Lobbezoo et al. [21]: self-reported grinding/snapping at night and clenching during the day in combination with clinical examination grading of tooth wear facets using BEWE index, masseter hypertrophy, linea alba and scalloped tongue edges. Individuals who presented both self-reported bruxism and clinical signs were classified as definite bruxism cases and those presenting only one criterion were classified as probable bruxism cases. Definite bruxism and probable bruxism groups were combined into bruxism group for further analyses.

### Assessment of psychological stress and sleep quality

Subjective stress was assessed using the 10-item Perceived Stress Scale (PSS-10), in which each item is scored 0–4 (never to very often) and summed to a total range of 0–40, with higher scores indicating greater perceived stress; scores ≥20 were operationally defined as high stress for this study, consistent with prior noise-exposure literature. Subjective sleep quality was measured using the 19-item self-rated Pittsburgh Sleep Quality Index (PSQI), which yields seven component scores (0–3 each: subjective sleep quality, sleep latency, sleep duration, habitual sleep efficiency, sleep disturbances, use of sleep medication, daytime dysfunction) summed to a global score of 0–21; scores >5 were defined as poor sleep quality [22]. Both scales were administered by trained research coordinator.

### Statistical analysis

Statistical analysis was performed using a licensed version of IBM SPSS Statistics v26.0. Normality was checked with the Shapiro–Wilk test and variance homogeneity with Levene’s test (all p > 0.05). Categorical variables were compared using chi-square, continuous variables using Student’s t-test, and the ordinal VAS scale using the Mann–Whitney U test with effect size r. A multivariable binary logistic regression (TMD as outcome) included noise exposure, bruxism, malocclusion, sex, PSS-10, PSQI, and PHQ-4, chosen a priori for clinical relevance. Multicollinearity was checked via VIF (all VIF < 2.0, tolerance > 0.5). Model fit was assessed with the Hosmer–Lemeshow test and Nagelkerke R^2^Significance was set at α < 0.05. logistic regression and mediation analysis were conducted to address distinct, complementary questions: logistic regression identified independent predictors of TMD and quantified their adjusted association after accounting for confounders, whereas medication analysis explored the extent to which the noise TMD association may operate through hypothesized psychosocial and behavioural pathways (bruxism and perceived stress). Mediation analysis used a bootstrapped (5000 resamples) product of coefficients approach; given the simultaneous cross-sectional measurement of exposure, mediators, and outcomes, results are interpreted as evidence of statistical medication rather than confirmed causal pathways. Odds ratios were used as the primary measure of association because the multivariable analysis was conducted using binary logistic regression, the standard approach for adjusting simultaneously for multiple categorical and continuous covariates in this study design. However, given the high prevalence of TMD in both groups (61.3% and 25.3%), the odds ratio likely overestimates the true prevalence ratio; this should be considered when interpreting the magnitude, though not the direction, of the observed associations.

## Results

There were no missing data for demographic variables, noise measurements or primary outcome measures (TMD, bruxism, malocclusion). Less than 1% of participants had missing data on the psychosocial scales (PSS-10, PSQI, PHQ-4; n = 2–3 per scale) and it was equally distributed between groups.

Baseline characteristics and noise exposure data are shown in Table 1. Groups were well-balanced for all demographic variables (all p > 0.05). Noise exposure was significantly greater in high-traffic residents (74.6 ± 6.8 dBA vs. 52.3 ± 5.1 dBA; mean difference 22.3 dBA; 95% CI: 20.9–23.7; p < 0.001). Participants assigned to the high-traffic area also reported significantly higher perceived stress (PSS-10: 22.4 ± 5.3 vs. 14.8 ± 4.7; p < 0.001) and worse sleep quality (PSQI: 8.1 ± 3.2 vs. 4.6 ± 2.8; p < 0.001), supporting our hypothesised stress-mediation hypothesis. These two psychosocial measures were therefore carried forward as covariates in our multivariable model.

**Table 1:** Baseline characteristics of study participants by area of residence (n = 300).

Characteristic	High-Traffic Area (n = 150)	Residential Area (n = 150)	p-value
Age, years — mean ± SD	34.6 ± 9.2	33.9 ± 8.7	**0.48**
**Sex**			
Male	72 (48.0%)	73 (48.7%)	0.90
Female	78 (52.0%)	77 (51.3%)	
**Education Level**			
Primary/secondary	61 (40.7%)	58 (38.7%)	0.71
Tertiary	89 (59.3%)	92 (61.3%)	
**Employment status**			
Employed	118 (78.7%)	114 (76.0%)	0.57
Unemployed/student	32 (21.3%)	36 (24.0%)	
Years of residence-mean ± SD	7.4 ± 4.1	6.9 ± 3.8	0.27
**Noise exposure**			
Mean LAeq,24h (dBA) ± SD	74.6 ± 6.8	52.3 ± 5.1	<0.001
Mean difference (95% CI)	22.3 (20.9–23.7)	–	<0.001
High exposure >65 dBA, n (%)	117 (78.0%)	33 (22.0%)	<0.001
**Psychosocial measures**			
PSS-10 Mean ± SD	22.4 ± 5.3	14.8 ± 4.7	<0.001
High stress (PSS-10 ≥ 20), n (%)	98 (65.3%)	41 (27.3%)	<0.001
PSQI mean ± SD	8.1 ± 3.2	4.6 ± 2.8	<0.001
Poor sleep (PSQI >5), n (%)	104 (69.3%)	52 (34.7%)	<0.001

*Data presented as n (%) or mean ± SD. p-values from independent samples t-test (continuous) or Pearson chi-square (categorical). PSS-10 = Perceived Stress Scale-10; PSQI = Pittsburgh Sleep Quality Index; BMI = body mass index; LAeq,24h = energy-averaged 24-hour noise level.*

Table 2 displays descriptive statistics for all clinical oral health outcomes by study group. On all assessed outcomes, individuals residing near high traffic experienced significantly worse oral health. TMJ problems were reported by 61.3% of those assigned to the high-traffic group compared to 25.3% of low traffic area residents (OR = 4.68; 95% CI: 2.84–7.71; p < 0.001). On the Helkimo severity grading scale, higher grades indicative of worsening TMJ dysfunction were progressively more frequent in the high-traffic group, χ^2^ = 40.50, p < 0.001, such that moderate and severe dysfunction combined was seen in 32.0% of high traffic participants compared with 10.6% assigned to reside near low traffic (χ^2^ = 40.50; p < 0.001). Malocclusion was twice as frequent in the high-traffic group (52.0% vs. 26.0%; χ^2^ = 21.31; p < 0.001) and bruxism was also significantly more prevalent (42.7% vs. 14.0%; χ^2^ = 30.35; p < 0.001). Median TMJ pain intensity was also significantly greater in those assigned to the high-traffic group (median VAS 4 [IQR 2–7] vs. 0 [IQR 0–3]; effect size r = 0.46; p = 0.001), a clinically meaningful difference that approached the threshold for moderate pain.

**Table 2:** Clinical oral health outcomes by area of Residence (n = 300).

Outcome	High-Traffic Area (n = 150)	Residential Area (n = 150)	OR (95% CI)/ Statistic	p-value
**TMJ symptoms present**	92 (61.3%)	38 (25.3%)	OR = 4.68 (2.84–7.71)	<0.001
**TMJ dysfunction severity (Helkimo)**			χ² = 40.50	<0.001
None	58 (38.7%)	112 (74.7%)	—	
Mild	44 (29.3%)	22 (14.7%)	—	
Moderate	32 (21.3%)	11 (7.3%)	—	
Severe	16 (10.7%)	5 (3.3%)	—	
**Malocclusion present**	78 (52.0%)	39 (26.0%)	χ² = 21.31	<0.001
**Bruxism present**	64 (42.7%)	21 (14.0%)	χ² = 30.35	<0.001
**TMJ pain — Median VAS (IQR)**	4 (2–7)	0 (0–3)	r = 0.46	0.001
No pain (VAS = 0)	58 (38.7%)	112 (74.7%)	—	
Mild (VAS 1–3)	28 (18.7%)	14 (9.3%)	—	
Moderate (VAS 4–6)	36 (24.0%)	15 (10.0%)	—	
Severe (VAS 7–10)	28 (18.7%)	9 (6.0%)	—	

*OR = odds ratio; CI = confidence interval; VAS = Visual Analogue Scale; r = effect size from Mann–Whitney U test. Helkimo Index: Di 0 = none; Di I = mild; Di II = moderate; Di III = severe (supplementary severity grading; primary TMD diagnosis by DC/TMD Axis I). Bruxism classified per Lobbezoo et al.* [17]*; definite and probable combined as present.*

As displayed in Table 3, There was a strong dose-response trend between noise exposure tertiles and TMD prevalence (χ^2^ for trend = 18.64; p < 0.001). TMD prevalence increased monotonically from 28.0% in the lowest tertile to 46.0% in the moderate tertile to 56.0% in the highest tertile (ORs of 2.18 and 3.27 respectively, compared to the lowest tertile). This monotonic trend is consistent with a dose response pattern and is compatible with the biological gradient criterion of Hill’s Criteria [23].

**Table 3:** TMD prevalence by noise exposure tertile — Dose-response analysis (n = 300).

Noise Exposure Tertile	LAeq,24h Range (dBA)	n	TMD Present n (%)	OR vs. Tertile 1 (95% CI)	p-value
Low (Tertile 1)	42.0–54.9	100	28 (28.0%)	Reference	—
Moderate (Tertile 2)	55.0–67.9	100	46 (46.0%)	2.18 (1.22–3.91)	0.008
High (Tertile 3)	68.0–88.5	100	56 (56.0%)	3.27 (1.83–5.84)	<0.001

*χ*^2^ *for trend = 18.64, p < 0.001. OR = odds ratio; CI = confidence interval. Reference = Tertile 1.*

As seen in Table 4, The final multivariable-adjusted model showed good calibration (Hosmer–Lemeshow goodness-of-fit p = 0.52) and moderate overall predictive ability (Nagelkerke R^2^ = 0.44). Compared to participants who had low/no noise exposure, participants who had noise exposures >65 dBA had the greatest odds of TMD (aOR = 3.10; 95% CI: 2.00–4.80; p < 0.001), independent of all psychosocial and clinical covariates. Fully-adjusted significant odds of TMD were also found for bruxism (aOR = 2.60), malocclusion (aOR = 2.00), perceived stress (aOR = 1.80), anxiety/depression (aOR = 1.70), poor sleep (aOR = 1.60), and female sex (aOR = 1.50). As a sensitivity analysis, the model was re-estimated after excluding 6 participants identified as outliers based on Cook’s distance (>4/n); results were materially unchanged (aOR for high noise exposure = 3.02, 95% CI: 1.94–4.71). Additionally, excluding 4 participants with extreme noise exposure (LAeq,24h ≥ 3 SD from the mean) did not materially alter the findings (aOR = 3.15, 95% CI: 2.03–4.89).

**Table 4:** Multivariable logistic regression: Independent predictors of TMD (n = 300). Outcome: TMD present vs. absent (DC/TMD Axis I) Model fit: Hosmer–Lemeshow χ^2^ = 7.12, p = 0.52 | Nagelkerke R^2^ = 0.44.

Risk Factor	aOR	95% CI	p-value
High noise exposure (>65 dBA)	3.10	2.00–4.80	<0.001
Bruxism (present vs. absent)	2.60	1.70–3.90	<0.001
Malocclusion (present vs. absent)	2.00	1.30–3.10	0.002
High perceived stress (PSS-10 ≥ 20)	1.80	1.20–2.80	0.006
Anxiety/depression (PHQ-4 ≥ 6)	1.70	1.10–2.60	0.014
Poor sleep quality (PSQI >5)	1.60	1.10–2.50	0.020
Female sex (vs. male)	1.50	1.00–2.30	0.046

*All variables entered simultaneously. Reference categories: low noise (≤65 dBA), bruxism absent, normal occlusion, PSS-10 < 20, PHQ-4 < 6, PSQI ≤5, male sex. aOR = adjusted odds ratio; CI = confidence interval; DC/TMD = Diagnostic Criteria for Temporomandibular Disorders; PSS-10 = Perceived Stress Scale-10; PSQI = Pittsburgh Sleep Quality Index; PHQ-4 = Patient Health Questionnaire-4.*

As presented in Table 5, When modeled together, bruxism and perceived stress accounted for 41.4% of the total association between noise and TMD (indirect effect β = 0.24; bootstrapped 95% CI: 0.15–0.33; p < 0.001), a pattern compatible with mediation, though the cross sectional design cannot establish temporal precedence among exposure, mediators and outcome. Indirect effects were larger for bruxism (β = 0.14; 24% of total effect) compared to perceived stress (β = 0.10; 17% of total effect). The direct effect of noise exposure remained strong and significant after adjusting for both bruxism and perceived stress (β = 0.34; 95% CI: 0.19–0.49; p < 0.001), which accounted for the remaining 58.6% of the total effect of noise on TMD.

**Table 5:** Mediation analysis: Pathways from noise exposure to TMD (n = 300).

Pathway	β coefficient	Bootstrapped 95% CI	p-value
Total effect: noise → TMD	0.58	0.41–0.75	<0.001
Direct effect: noise → TMD (controlling mediators)	0.34	0.19–0.49	<0.001
Indirect via bruxism	0.14	0.08–0.22	<0.001
Indirect via perceived stress	0.10	0.04–0.18	0.002
Total indirect (mediated) effect	0.24	0.15–0.33	<0.001
Proportion mediated	41.4%	—	—

*Bootstrapped CIs based on 5,000 resamples. both indirect pathways excluded zero, consistent with potential mediation. As exposure, mediators, and outcome were assessed concurrently in this cross sectional design, these results cannot establish temporal precedence or confirm causal mediation.*

## Discussion

This study makes two important contributions to current knowledge on environmental noise and oral health. First, our results provide novel population-level evidence that chronic environmental noise exposure is independently associated with increased odds of TMD, bruxism, and malocclusion in an asymptomatic community-based cohort of adults living in New Delhi, India. Residents of high traffic residences experienced significantly greater odds of TMJ symptoms (61.3% vs. 25.3%), malocclusion (52.0% vs. 26.0%), and bruxism (42.7% vs. 14.0%). Compared to low traffic area residents, these differences were significant across all outcomes. In addition, after adjusting for potential confounders and mediators, high noise exposure was associated with the greatest odds of TMD of any variable included in the multivariable model (aOR = 3.10; 95% CI: 2.00–4.80). This relationship was further supported by a dose response pattern; prevalence of TMD rose monotonically as tertials of noise exposure increased (28.0% → 46.0% → 56.0%; χ^2^ for trend p < 0.001). While a biological gradient is one feature often considered supportive of causal relationships, this observation alone is insufficient to establish in a cross-sectional design.

### Biological mechanism

Theoretically, these results can be explained by a neuroendocrine stress response pathway that has been well established in animal models and supported by human studies. Noise-induced activation of the HPA axis is associated with an increase in circulating cortisol and catecholamines, potentially associated with heightened muscle tonus of the masticatory muscles [12]. Chronically elevated muscle tonus may be associated with bruxism, which further loads the TMJ and is associated with tooth grinding through accelerated occlusal wear [13,14]. Mediation analysis showed bruxism and perceived stress jointly mediated 41.4% of the total association between noise exposure and TMD (Natural Indirect Effect = 41.4%, 95% CI: 29.1–55.6%), with bruxism as the dominant mediator (24%). As exposure, mediators and outcome were all measured simultaneously in this cross sectional design, temporal precedence among these variable cannot be established; these findings are therefore compatible with, but not proof of, a mediating pathway. Of note, the majority of the risk was retained directly by the noise-exposure pathway (58.6%; abstinence mediated effect), indicating noise-related influences on TMD arise from pathophysiological mechanisms beyond psychologic arousal (i.e., direct acoustic-vibratory stimulation of periarticular structures in the TMJ [14], or possibly systemic inflammation independent of HPA axis activation [12]). These findings were further substantiated by the extremely high prevalence of bruxism observed in the high traffic group compared to published population norms of 8–13% for sleep bruxism and 22–30% for awake bruxism [16,21]. In sum, chronic noise was an upstream risk indicator for bruxism.

### Psychosocial pathways

Adjusted odds of TMD were greater in respondents who reported high perceived stress (aOR = 1.80), poor sleep quality (aOR = 1.60), or anxiety/depression (aOR = 1.70), lending support to prior studies by Pavlou et al. [16] and Arregi et al. [14] which demonstrated that road traffic noise causes sustained sympathetic nervous system activation and sleep disturbance regardless of subjective stress perception. We found significantly higher scores on both the PSS-10 and PSQI in participants living on high traffic roads compared to their low traffic counterparts, indicating stress and sleep disturbance mediate the relationship between noise exposure and TMD risk rather than serving as proxy measures for noise.

### Bidirectionality and confounding

Reverse causation cannot be ruled out with this cross-sectional study design. Pain sensitisation as a consequence of TMD may increase perceived annoyance from noise and sleep disturbance due to TMD may increase noise sensitivity creating a vicious cycle. Traffic-heavy areas may also be subject to co-exposures correlated with noise such as air pollution, socioeconomic deprivation, occupational noise exposures, all of which could play a role in TMD risk. The higher prevalence of malocclusion observed in the high traffic group likely reflects pre-existing skeletal or occlusal characteristics rather than a causal effect of noise exposure, and should not be interpreted as evidence that environmental noise drives malocclusion development. Confounding by these and other unmeasured factors is possible despite adjustment for known confounders in the multivariable model [7].

### Sex differences

Female sex remained significantly associated with TMD in the full adjusted model (aOR = 1.50), as has been shown previously and is likely due to hormonal effects on ligament looseness and sensitisation of pain pathways. The unadjusted analysis was not significant (p = 0.11), however appeared after controlling for noise exposure and bruxism in the multivariable analysis. This highlights that sex-based differences in disease risk may be dependent on other environmental and behavioural factors.

### Clinical and public health implications

Noise should be considered as a potential risk factor for TMD in dental clinical settings, especially in high traffic areas. Dentists should be aware of bruxism as a symptom and screen their patients accordingly. Public health initiatives aimed at noise reduction should consider oral health as a co-objective when planning noise mitigation strategies such as rerouting traffic, sound proofing residences and implementing green space buffers. Noise reduction could have many benefits for cardiovascular health, sleep disturbance, and as demonstrated here, TMD.

### Limitations

This study has several limitations: the cross-sectional design precludes causal inference; non-probability sampling from a single neighbourhood per group in one city limits external validity; residual confounding (air pollution, occupation, income, safety, healthcare access, population density) could not be excluded; the short-duration noise assessment does not fully confirm long-term acoustic stability; bruxism relied on self-report rather than polysomnography/electromyography; occlusal assessment was limited to Angle’s classification; and findings are limited to one city and a five-day recruitment window. Additionally, given the high prevalence of the primary outcome (TMD) in both exposure groups, the odds ratios reported likely overestimate the corresponding prevalence ratios; a Poisson regression with robust variance or a log-binomial model would have yielded a prevalence ratio better suited to a cross-sectional design with a common outcome, and future studies should consider this approach.

## Conclusion

Our study provides novel evidence that chronic high environmental noise exposure is associated with a higher prevalence of TMD, bruxism, and malocclusion among urban adults, with a dose dependent pattern across noise exposure tertiles. Given the cross-sectional design, these findings should be interpreted as an association rather than evidence of causality. Exposure to chronic high environmental noise levels (>65 dBA) was found to be the strongest independent risk factor for TMD (aOR = 3.10), and bruxism and perceived stress were shown to partially mediate the effect of high noise exposure on TMD risk. Study findings support previous hypotheses of a biologically plausible noise–stress–parafunctional activity pathway leading to orofacial pain and dysfunction. Dental clinicians should consider bruxism/TMD screening among patients who frequent high traffic noise environments and public health agencies should begin to include oral health outcome measures in the burden of urban environmental noise. Prospective longitudinal studies that utilize objective noise dosimetry and DC/TMD criteria are needed to further investigate causal relationships.

## Supporting information

S1 File STROBE checklist. (DOC)

## References

[1] Jamir L, Nongkynrih B, Gupta SK. Community noise pollution in urban India: Need for public health action. Indian J Community Med. 2014;39(1):8–12. doi: 10.4103/0970-0218.126342 24696533 PMC3968587

[2] de Paiva Vianna KM, Alves Cardoso MR, Rodrigues RMC. Noise pollution and annoyance: An urban soundscapes study. Noise Health. 2015;17(76):125–33. doi: 10.4103/1463-1741.155833 25913551 PMC4918656

[3] Tabaei S, Rashki Ghalenoo S, Panahandeh M, Bagheri G, Tabaee SS. The relationship between noise pollution and cardiovascular diseases: an umbrella review on meta-analyses. BMC Cardiovasc Disord. 2025;25(1):630. doi: 10.1186/s12872-025-04864-9 40859126 PMC12379546

[4] Soman C, Faisal AT, Alsaeygh MM, Al Saffan AD, Salma RG. Driving stress-induced effects on the orofacial region and its functions and health behaviors in Riyadh: A cross-sectional survey. Healthcare (Basel). 2024;12(15):1538. doi: 10.3390/healthcare12151538 39120241 PMC11311540

[5] Vitazkova D, Kosnacova H, Turonova D, Foltan E, Jagelka M, Berki M, et al. Transforming Sleep Monitoring: Review of Wearable and Remote Devices Advancing Home Polysomnography and Their Role in Predicting Neurological Disorders. Biosensors. 2025;15(2):117. doi: 10.3390/bios15020117 PMC11853583 39997019

[6] Petri D, Licitra G, Vigotti MA, Fredianelli L. Effects of exposure to road, railway, airport and recreational noise on blood pressure and hypertension. Int J Environ Res Public Health. 2021;18(17):9145. doi: 10.3390/ijerph18179145 34501735 PMC8431620

[7] Basner M, Babisch W, Davis A, Brink M, Clark C, Janssen S, et al. Auditory and non-auditory effects of noise on health. Lancet. 2014;383(9925):1325–32. doi: 10.1016/S0140-6736(13)61613-X 24183105 PMC3988259

[8] Clark C, Crumpler C, Notley H. Evidence for environmental noise effects on health for the United Kingdom policy context: A systematic review of the effects of environmental noise on mental health, wellbeing, quality of life, cancer, dementia, birth, reproductive outcomes, and cognition. Int J Environ Res Public Health. 2020;17:393. doi: 10.3390/ijerph17020393 PMC7013411 31936110

[9] Lile IE, Hajaj T, Constantin GD, Niculescu ST, Marian D, Stana O, et al. Association between occlusal interferences, temporomandibular joint dysfunction, and bruxism in romanian adults. J Clin Med. 2025;14(16):5612. doi: 10.3390/jcm14165612 40869440 PMC12386957

[10] Lekaviciute R, Kriauciunas A. Relationship between occlusal factors and temporomandibular disorders: A systematic literature review. Cureus. 2024;16(2):e54130. doi: 10.7759/cureus.54130 38487145 PMC10939299

[11] Jogna F, Graenicher A A, Rey-Millet Q, Groz A, De Grasset J, Stollar F, et al. Pharmacological and non-pharmacological approaches to temporomandibular disorder chronic pain: A narrative review. Pain Manag. 2025;15(5):285–96. doi: 10.1080/17581869.2025.2502311 40338653 PMC12118397

[12] Hahad O, Prochaska JH, Daiber A, Münzel T. Environmental noise-induced effects on stress hormones, oxidative stress, and vascular dysfunction: Key factors in the relationship between cerebrocardiovascular and psychological disorders. Oxid Med Cell Longev. 2019;2019:1–13. doi: 10.1155/2019/4623109 PMC6878772 31814877

[13] Reddy SV, Kumar MP, Sravanthi D, Mohsin AHB, Anuhya V. Bruxism: A literature review. J Int Oral Health. 2014;6(6):105–9. 25628497 PMC4295445

[14] Arregi A, Vegas O, Lertxundi A, Silva A, Ferreira I, Bereziartua A, et al. Road traffic noise exposure and its impact on health: evidence from animal and human studies-chronic stress, inflammation, and oxidative stress as key components of the complex downstream pathway underlying noise-induced non-auditory health effects. Environ Sci Pollut Res Int. 2024;31(34):46820–39. doi: 10.1007/s11356-024-33973-9 38977550 PMC11297122

[15] Jakovljević B, Belojević G, Paunović K, Stojanov V. Road traffic noise and sleep disturbances in an urban population: cross-sectional study. Croat Med J. 2006;47(1):125–33. 16489705 PMC2080382

[16] Pavlou IA, Spandidos DA, Zoumpourlis V, Papakosta VK. Neurobiology of bruxism: The impact of stress (Review). Biomed Rep. 2024;20(4):59. doi: 10.3892/br.2024.1747 38414628 PMC10895390

[17] Chauhan BS, Kumar S, Garg N, Gautam C. Evaluation and analysis of environmental noise levels in NCT of Delhi, India. MAPAN. 2023;38(2):409–29. doi: 10.1007/s12647-022-00620-y

[18] von Elm E, Altman DG, Egger M, Pocock SJ, Gøtzsche PC, Vandenbroucke JP, et al. Strengthening the reporting of observational studies in epidemiology (STROBE) statement: Guidelines for reporting observational studies. BMJ. 2007;335(7624):806–8. doi: 10.1136/bmj.39335.541782.AD 17947786 PMC2034723

[19] World Health Organization. Environmental Noise Guidelines for the European Region. 2018.

[20] Schiffman E, Ohrbach R, Truelove E, Look J, Anderson G, Goulet J-P, et al. Diagnostic criteria for temporomandibular disorders (DC/TMD) for clinical and research applications: Recommendations of the International RDC/TMD Consortium Network* and Orofacial Pain Special Interest Group†. J Oral Facial Pain Headache. 2014;28(1):6–27. doi: 10.11607/jop.1151 24482784 PMC4478082

[21] Lobbezoo F, Ahlberg J, Glaros AG, Kato T, Koyano K, Lavigne GJ, et al. Bruxism defined and graded: an international consensus. J Oral Rehabil. 2013;40(1):2–4. doi: 10.1111/joor.12011 23121262

[22] Manfredini D, Lobbezoo F. Role of psychosocial factors in the etiology of bruxism. J Orofac Pain. 2009;23(2):153–66. 19492540

[23] Hill AB. The environment and disease: association or causation? Proc R Soc Med. 1965;58: 295–300. doi: 10.1177/003591576505800503 PMC1898525 14283879

